# Replication of West Nile virus, Rabensburg lineage in mammalian cells is restricted by temperature

**DOI:** 10.1186/1756-3305-5-293

**Published:** 2012-12-14

**Authors:** Matthew T Aliota, Laura D Kramer

**Affiliations:** 1 The Arbovirus Laboratories, Wadsworth Center, New York State Department of Health, 5668 State Farm Rd., Slingerlands, NY 12159, USA; 2 Department of Biomedical Sciences, School of Public Health, State University of New York at Albany, Albany, NY, USA

**Keywords:** Arbovirus, Host Range, Temperature, Insect-specific Flavivirus, West Nile virus, Rabensburg virus

## Abstract

**Background:**

The genus *Flavivirus* currently consists of approximately 80 single-strand positive-sense RNA viruses. These replicate in a range of hosts including myriad vertebrate, insect, and tick species. As a consequence of this broad host range, the majority of flaviviruses can be propagated in most vertebrate and insect cell cultures. This ability to infect arthropods and vertebrates usually is essential for maintenance of these viruses in nature. But recently, there has been the discovery of a number of flaviviruses that infect mosquitoes but not vertebrates. It remains largely unknown why certain flaviviruses infect vertebrates and mosquitoes while others infect mosquitoes or vertebrates exclusively.

**Methods:**

Here, we initiated *in vitro* host range studies of Rabensburg virus (RABV), an intermediate between the mosquito-specific and horizontally transmitted flaviviruses, to provide information on the factor(s) that underlie the varying host range of flaviviruses. RABV is an intermediate between the mosquito-specific and horizontally transmitted flaviviruses because it does not infect mammalian or avian cell cultures, house sparrows, or chickens, but it does share genetic characteristics with the Japanese Encephalitis serogroup of flaviviruses.

**Results:**

*In vitro* growth kinetic assays revealed the complete abrogation of RABV growth on Vero and E6 cells incubated at temperatures 35°C and higher, but surprisingly RABV infected, replicated efficiently, and displayed overt cytopathic effects (CPE) on Vero and E6 cell cultures incubated below 35°C. In contrast, RABV was fully viable, replicated efficiently, and displayed overt CPE on C6/36 cells incubated at 28°C or 37°C, thus implicating temperature as an important factor limiting the host range of RABV.

**Conclusions:**

These data are critical for further study to more fully identify the determinants that mediate the evolution of biological transmission among flaviviruses. It also will be useful for studies that look to provide a comprehensive molecular definition of flavivirus-host cell interactions. And it will provide a cadre of information to design wet lab experiments to investigate the genetic changes that facilitate host switching, which may lead to new vertebrate pathogens or transmission pathways.

## Background

The genus *Flavivirus* (family *Flaviviridae*) presently comprises approximately 80 single-strand positive-sense RNA viruses, and consists of four groups, each with a distinct host range. The four groups include insect (mosquito) viruses without the capacity to replicate in vertebrates, vertebrate viruses without a vector (no known vector, NKV), tick-borne viruses that replicate in ticks and vertebrates, and mosquito-borne viruses that replicate in mosquitoes and vertebrates [1]. Despite the presence of host-restricted groups within the genus, the majority of flaviviruses are transmitted horizontally between vertebrate hosts and hematophagous arthropods, such as mosquitoes and ticks. In fact, theoretical studies have estimated the existence of over 2000 undiscovered mosquito-borne flaviviruses [2]. And several members of the genus, such as *Dengue virus* (DENV), *Yellow fever virus* (YFV), *West Nile virus* (WNV), *Japanese encephalitis virus* (JEV), and *Tick-borne encephalitis virus* (TBEV), are human pathogens that represent significant global health problems [3]. Recently, there has been an upsurge in the discovery of ‘insect-specific’ flaviviruses and/or their related sequences in natural mosquito populations. Examples include Culex flavivirus (CxFV), Cell fusing agent virus (CFAV), Quang Binh virus, Kamiti River virus (KRV), and Aedes flavivirus, among others [4-7]. Yet, our understanding of the significance of the ‘insect-specific’ flaviviruses and the implications for the evolution and transmission of viruses belonging to the genus *Flavivirus* currently is limited.

Current dogma suggests that the ‘insect specific’ and ‘traditional’ vector-borne flaviviruses form sister groups which are both highly divergent from the other members of the family *Flaviviridae*[8]. Several studies have hypothesized that the vector-borne group of flaviviruses evolved from a group of vertebrate viruses (NKV group), i.e., the absence of a vector is the ancestral condition for this family of viruses [8-12]. In contrast, it has been hypothesized that the NKV group evolved from insect-specific viruses (e.g., CFAV and KRV) or from a no-vector vertebrate virus (e.g., Tamana bat virus) [13], although this is somewhat controversial and surely requires further study [8]. Regardless, it would be reasonable to hypothesize that at least some of the current group of horizontally transmitted flaviviruses evolved from insect-specific viruses and it also would be reasonable to assume that some of these newly discovered mosquito-specific viruses have the potential to emerge and adapt to new host environments, i.e., humans or other vertebrates.

Recently, we characterized an intermediate between the mosquito-specific and horizontally transmitted flaviviruses, Rabensburg virus (RABV; prototype strain 97–103) [14]. RABV is a *Flavivirus* with ~76% nucleotide and 90% amino acid identity with representative members of lineage one and two WNV [15]. However, RABV97-103 did not infect mammalian or avian cell cultures, house sparrows or chickens, but the virus efficiently infected mosquito cells. In addition, mosquitoes within the *Culex pipiens* complex supported replication of RABV but displayed poor peroral vector competence for this virus as compared to wild type WNV, and the same mosquitoes vertically transmitted the virus at a much higher rate than what had been reported for wild type WNV [14]. Therefore, RABV could be used as a model to provide significant insight into the determinants of flavivirus attenuation in vertebrates and could increase our understanding of the link between the ‘insect-specific’ flaviviruses and those that are transmitted between mosquitoes and vertebrates, further clarifying the evolution of flaviviruses.

Accordingly, we initiated *in vitro* host range studies of RABV97-103 and the most recent isolate, RABV strain 06–222 [16], to provide information on the factor(s) that underlie the varying host range of flaviviruses. It remains largely unknown which viral determinants are responsible for host cell tropism and vector specificity, i.e., why do certain flaviviruses infect mosquitoes and vertebrates and why are other flaviviruses not able to infect vertebrates or vertebrates exclusively? Here, we demonstrate that the factor limiting the ability of RABV to infect mammalian cell culture is temperature, and to our knowledge, this is the first demonstration of a member of the genus *Flavivirus* exhibiting a narrow host range as a result of temperature sensitivity.

## Methods

### Cells

African Green Monkey kidney cells (Vero; ATCC #CCL-81) and a clone of standard Vero cells (E6; ATCC #CRL-1586) were grown in minimal essential medium (MEM; Gibco, Carlsbad, CA) supplemented with 10% fetal bovine serum (FBS; Hyclone, Logan, UT), 2 mM L-glutamine, 1.5 g/l sodium bicarbonate, 100 U/ml of penicillin, 100 μg/ml of streptomycin, and incubated at 37°C in 5% CO_2_. *Aedes albopictus* mosquito cells, (C6/36; ATCC #CRL-1660) were maintained in MEM supplemented with 10% FBS, 2 mM L-glutamine, 1.5 g/l sodium bicarbonate, 0.1 mM non-essential amino acids, 100 U/ml of penicillin, 100 μg/ml of streptomycin, and incubated at 28°C in 5% CO_2_. *Culex tarsalis* mosquito cells (CxT; courtesy of Aaron Brault, Centers for Disease Control and Prevention, Ft. Collins, CO, USA) were maintained in Schneider’s Insect Media (Sigma-Aldrich Inc., St. Louis, MO) supplemented with 10% FBS, and incubated at 28°C in 5% CO_2_.

### Viruses

RABV isolate 97–103 [GenBank AY765264], originally isolated from *Cx. pipiens* in the Czech Republic, and RABV isolate 06–222 [GenBank:GQ421359], originally isolated from *Aedes rossicus* in the Czech Republic, were obtained from Zdenek Hubalek (Institute of Vertebrate Biology, Academy of Sciences, Brno, Czech Republic). Virus stocks were prepared by inoculation onto a confluent monolayer of C6/36 mosquito cells and a clarified harvest of the culture medium was collected after six days of incubation at 28°C. These stocks were titered by plaque assay on Vero cells at 28°C. The titers by plaque assay on Vero cells for RABV97-103 and RABV06-222 were log_10_ 7.34 plaque forming units (PFU)/ml) and log_10_6.30 PFU/ml, respectively. WNV isolate WN02-1956 [GenBank:AY590210] was isolated from the kidney of an American Crow collected in New York State and isolated on Vero cells, followed by a single round of amplification on C6/36 cells. The titer by plaque assay on Vero cells was log_10_8.6 PFU/ml.

### *In vitro* viral replication

Six-well plates containing confluent monolayers of C6/36, CxT, Vero, or E6 cells were infected with virus (RABV97-103, RABV06-222, or WN02-1956), in triplicate, at multiplicity of infection (MOI) of 0.01 PFU/ml. After one hour of adsorption at 28°C or 37º, the inoculum was removed and the cultures were washed three times. Fresh media were added, the mosquito cell cultures were incubated for seven days at 28°C and 37°C, and Vero and E6 cell cultures were incubated for 14 days at 28°C and 37°C, with aliquots removed daily, diluted 1:10 in culture media, and stored at −80°C. Viral titers at each time point were determined by plaque titration on Vero cells. The absence of RABV growth also was confirmed by RT-PCR using RABV specific primers and the Qiagen one-step RT-PCR kit. Cytopathic effects (CPE) were observed using phase contrast optics on a Zeiss Axiovert 25 inverted microscope at 20X magnification, and phase contrast micrographs were taken of infected and uninfected C6/36 cell monolayers using AxioVision Software (Carl Zeiss Microscopy, Germany). RABV infection and replication also were assessed on Vero cells incubated at 32°C, 33°C, 34°C, and 35°C to identify the temperature threshold that blocks productive infection.

### Plaque assay

Virus titration was performed on freshly confluent Vero cell monolayers in six-well plates. Duplicate wells were infected with 0.1 ml aliquots from serial 10-fold dilutions in growth media and virus was adsorbed for one hour. Following incubation, the inoculum was removed, and monolayers were overlaid with 3 ml containing a 1:1 mixture of 1.2% oxoid agar and 2X MEM (Gibco, Carlsbad, CA) with 10% (vol/vol) FBS and 2% (vol/vol) penicillin/streptomycin. Cells were incubated at 28°C in 5% CO_2_ for 10 days for RABV plaque development and incubated at 37°C in 5% CO_2_ for two days for WNV plaque development. Cell monolayers then were stained with 3 ml of overlay containing a 1:1 mixture of 1.2% oxoid agar and 2X MEM with 2% (vol/vol) FBS, 2% (vol/vol) penicillin/streptomycin, and 0.33% neutral red (Gibco). Cells were incubated overnight at 28° (RABV) or 37°C (WNV), plaques were counted, and RABV plaque morphology did not differ from WNV plaque morphology.

## Results and discussion

### Influence of temperature on virus growth on mammalian cells

Previously, we reported that RABV97-103 was unable to infect mammalian or avian cell cultures, house sparrows or chickens, but the virus efficiently infected mosquito cells [14], i.e., it displayed characteristics of a host-restricted flavivirus. The basis for its host-restriction occurred at the point of entry into mammalian cells, as infectious RABV97-103 RNA was able to replicate in human cells following transfection [14]. This was contradictory to a previous report that observed the production of overt CPE and replication of RABV97-103 on E6 and *Xenopus laevis* frog cells (XTC-2) [17]. It should be noted, however, that passage via intracranial inoculation of suckling mice had been required for RABV97-103 growth on E6 cells and that XTC-2 cells are cultured at 28°C. Subsequently, Hubalek et al. [15] showed that a second isolate of RABV (strain 06–222) produced CPE and replicated after original inoculation of mosquito suspension on E6 cells. These data suggested that there may be varying host range even among strains of RABV, which is consistent with the fact that RABV97-103 was isolated from a pool of *Cx. pipiens* and RABV06-222 was isolated from a pool of *Ae. rossicus*[16,17], yet no human isolate exists [18]. This, in conjunction with the fact that RABV97-103 replicated on mosquito cell culture and reports of RABV replicating on vertebrate cell culture at 28°C (e.g., XTC-2) but not on vertebrate cell culture at 37°C (e.g., Vero, avian, human), led us to postulate that temperature was an important determinant for RABV infection of mammalian cells.

**Figure 1:**
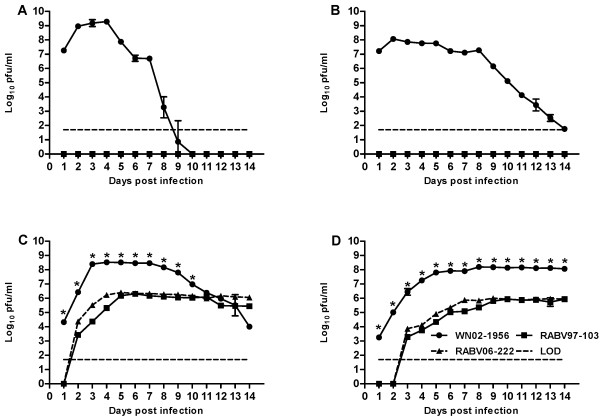
**Rabensburg virus grows on Vero and E6 cells incubated at 28°C but not at 37°C.** Data points represent means of three replicates at each time point +/− standard deviation. Cells were inoculated at a MOI of 0.01 PFU/ml. LOD, limit of detection; WN02-1956, West Nile virus strain WN02-1956; RABV97-103, Rabensburg virus prototype strain 97–103; RABV06-222, Rabensburg virus strain 06–222; pfu, plaque forming units; *, *p*<0.001. **A**.) Vero cells incubated at 37°C. **B**.) E6 cells incubated at 37°C. **C**.) Vero cells incubated at 28°C. **D**.) E6 cells incubated at 28°C.

**Figure 2:**
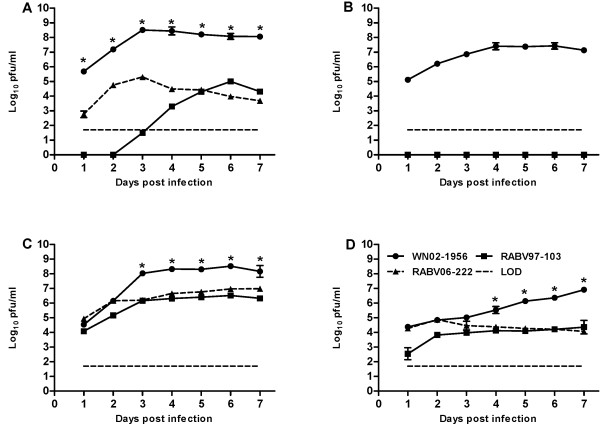
**Rabensburg virus grows on mosquito cells incubated at either 28°C or 37°C.** Data points represent means of three replicates at each time point +/− standard deviation. Cells were inoculated at a MOI of 0.01 PFU/ml. LOD, limit of detection; WN02-1956, West Nile virus strain WN02-1956; RABV97-103, Rabensburg virus prototype strain 97–103; RABV06-222, Rabensburg virus strain 06–222; pfu, plaque forming units; *, *p*<0.001. **A**.) *Aedes albopictus* (C6/36) cells incubated at 37°C. **B**.) *Culex tarsalis* cells incubated at 37°C. **C**.) *Aedes albopictus* (C6/36) cells incubated at 28°C. **D**.) *Culex tarsalis* cells incubated at 28°C.

In an effort to determine if temperature had any effect on the ability of RABV to infect Vero and E6 cell cultures, cells were inoculated at a MOI of 0.01 PFU/ml, and comparisons of growth kinetics of two RABV isolates (97–103 and 06–222) and wild-type WNV (WN02-1956) were made over the course of 14 days. Complete abrogation of RABV growth was observed on Vero and E6 cells at 37°C. In fact, it was concluded that the RABV isolates only grew on Vero and E6 cells at temperatures lower than 35°C, thus implicating temperature as an important determinant for RABV infection on mammalian cell culture. Data from representative experiments are depicted in Figures 1 and 2. The control virus, WN02-1956, replicated efficiently on Vero and E6 cells regardless of temperature (Figures 1A-D). In contrast, both RABV isolates were unable to infect Vero and E6 cell cultures at 37°C (Figure 1A and B). Surprisingly, both RABV isolates infected, replicated efficiently, and displayed overt CPE on Vero and E6 cell cultures incubated at 28°C (Figure 1C and D). At 28°C, both RABV isolates displayed significantly lower titers (Student’s *t*-test *p<* 0.001) as compared to WN02-1956. On Vero cells at 28°C, WN02-1956 reached a peak titer of 3.3 × 10^8^ PFU/ml four days post inoculation; whereas, RABV growth was below the limit of detection until two days post inoculation. And both RABV isolates did not reach peak titer (2.0 × 10^6^ PFU/ml) until six days post inoculation. Similarly, WN02-1956 replicated to a higher peak titer (6.3 × 10^7^ PFU/ml) in a shorter amount of time (five days post infection) as compared to both RABV isolates on E6 cells at 28°C (Figure 1C and D). RABV growth was below the limit of detection until three days post inoculation, and both RABV isolates did not reach peak titer (7 × 10^5^ PFU/ml) until seven days post inoculation on E6 cells at 28°C.

### Influence of temperature on virus growth on mosquito cells

**Figure 3:**
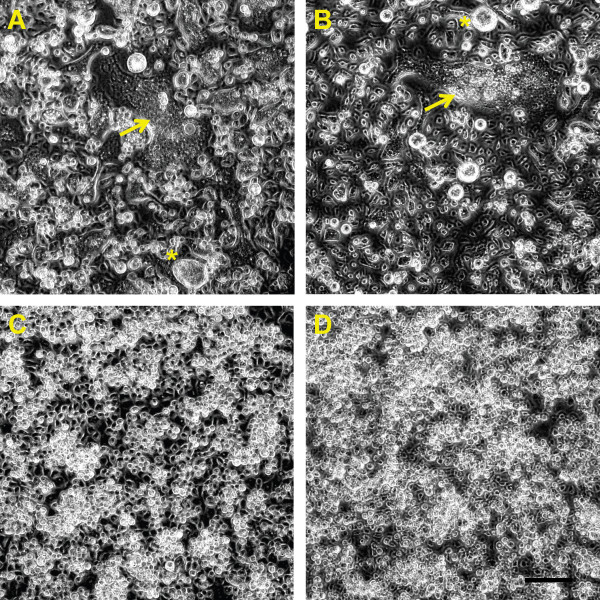
**Overt cytopathic effects associated with Rabensburg virus growth on *Aedes albopictus *cells incubated at 28°C.** At three days post inoculation extensive cell fusion, giant multi-nucleated cells, and syncytia formation are evident in C6/36 cells infected with Rabensburg virus strains 97–103 and 06–222 (**A**, **B**). No obvious pathology was observed in cells infected with West Nile virus strain WN02-1956 (**C**) or in mock inoculated cells (**D**) three days post inoculation. Cell fusion, yellow arrows; *, giant cells; A-D scale bar, 100 μm.

**Figure 4:**
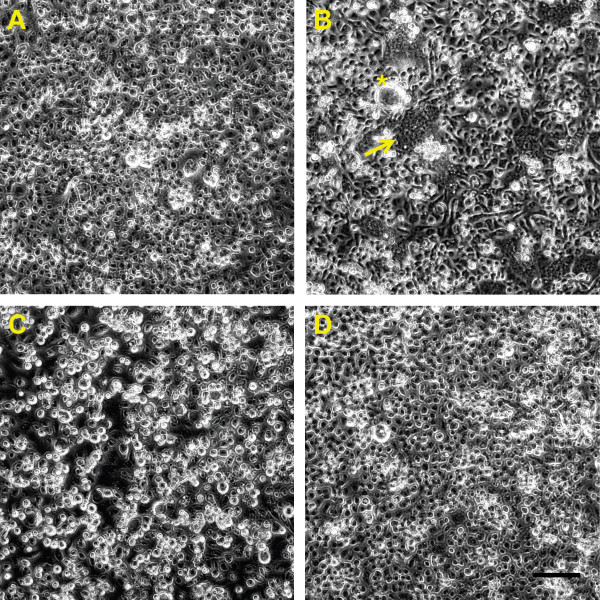
**Overt cytopathic effects associated with Rabensburg virus growth on *Aedes albopictus *cells incubated at 37°C.** At three days post inoculation no obvious pathology was observed in C6/36 cells infected with Rabensburg virus strain 97–103 (**A**), West Nile virus strain WN02-1956 (**C**), or in mock inoculated cells (**D**). Extensive cell fusion, giant multi-nucleated cells, and syncytia formation is evident in C6/36 cells infected with Rabensburg virus strain 06–222 (**B**) three days post inoculation. Cell fusion, yellow arrow; *giant multi-nucleated cell; A-D scale bar, 100 μm.

We then investigated whether temperature had a negative effect on RABV growth in mosquito cell culture. C6/36 and CxT cells were infected at a MOI of 0.01 PFU/ml, and comparisons of both RABV isolates and WNV were made over the course of seven days. All three viruses were fully viable, replicated efficiently, and displayed overt CPE on C6/36 cells incubated at 28°C (Figure 2C). CPE associated with both RABV isolates differed from CPE associated with WN02-1956 (Figure 3A-D). Both RABV isolates produced marked syncytia, giant multinucleated cells, and extensive cell fusion on C6/36 cells beginning at two days post inoculation. CPE was similar to that described previously with the prototype CFAV [19] and CxFV [20]. Interestingly, C6/36 cells inoculated with RABV97-103 and incubated at 37°C did not display overt CPE (Figure 4A), but the virus did replicate, albeit at a much slower rate than the two other viruses (Figure 2A). In contrast, C6/36 cells inoculated with RABV06-222 and incubated at 37°C displayed characteristic RABV CPE (Figure 4B) and replicated efficiently (Figure 2A). As expected, all three viruses also were fully viable and replicated on CxT cells incubated at 28°C; however, none of the viruses displayed overt CPE on this cell line (Figure 2D). Regardless of cell type or temperature, both RABV isolates replicated to significantly lower titers (Student’s *t*-test *p*<0.001) on mosquito cells as compared to WN02-1956. On C6/36 cells at 28°C and 37°C, WN02-1956 reached peak titers of 1.1 × 10^8^ and 3.2 × 10^8^ PFU/ml, respectively, three days post inoculation. Whereas, both RABV isolates only replicated to peak titers of ~3.2 × 10^6^ and ~2.0 × 10^5^ PFU/ml on C6/36 cells at 28°C and 37°C, respectively (Figure 2A and 2C). Similarly, on CxT cells at 28°C and 37°C, WN02-1956 replicated to peak titers of 8.1 × 10^6^ and 2.6 × 10^7^ PFU/ml, respectively. Both RABV isolates only replicated to peak titers of ~1.2 × 10^4^ PFU/ml on CxT cells at 28°C. RABV growth was below the plaque assay limit of detection at 37°C (Figure 2B and 2D); however, viral RNA was detected for both RABV isolates via RT-PCR using RABV specific primers, suggesting a low level of viral growth on this cell line incubated at 37°C (data not shown).

The factors that determine host restriction among flaviviruses are poorly understood, but the host range of any arbovirus unquestionably is governed by multiple abiotic and biologic factors including genetic traits of viruses and hosts for (review see [21,22]). For biological transmission to evolve, the factors that favor appropriate encounters among virus, competent vector, and susceptible vertebrate are of fundamental importance [13]. But, no matter how perfectly other basic requirements for the establishment of biological transmission are met, many viruses do not replicate sufficiently in certain hosts because of genetic constraints [13,23,24]. For example, available data suggest that mutations in the glycosylation patterns of the envelope and nonstructural protein 1 of certain flaviviruses can influence cell/tissue tropism, infectivity, and infection efficiency (e.g., [25-27]). However, these mutations result in attenuation and do not completely abolish replication. Until now, temperature has not been identified as a factor that can restrict flavivirus host range, although previous studies have suggested that alphavirus host range can be restricted, at least in part, by temperature. For example, *Sindbis virus* can be cultured to high titers from 15°C to 40°C [28,29]; whereas, *Salmon pancreas disease virus* has been demonstrated to have a narrow temperature range of 10°C to 15°C [30].

**Figure 5:**
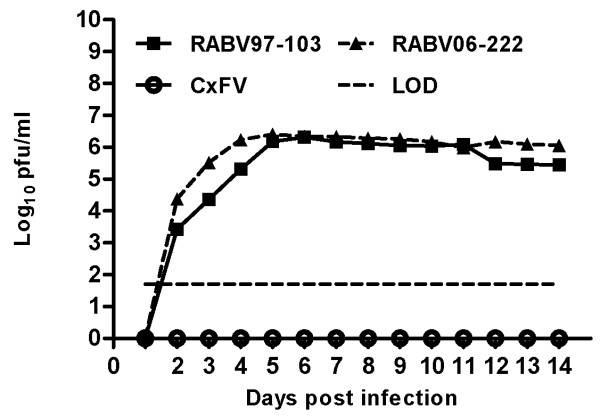
**Culex flavivirus (CxFV) does not grow on Vero cells incubated at 28°C.** Data points represent means of three replicates at each time point +/− standard deviation. Cells were inoculated at a MOI of 0.01 PFU/ml. The absence of CxFV growth was confirmed via RT-PCR using CxFV-specific primers. LOD, limit of detection; RABV97-103, Rabensburg virus prototype strain 97–103; RABV06-222, Rabensburg virus strain 06–222; CxFV, Culex flavivirus strain HOU24518; pfu, plaque forming units.

Here we demonstrated that temperatures of 35°C and above completely abolish replication of RABV on mammalian cell culture. The mechanism mediating this phenomenon is likely to be complex, and is not a panacea to explain host restriction among all insect-specific flaviviruses as CxFV [GenBank:FJ502995] still was not capable of infecting and replicating on Vero cells incubated at 28°C (Figure 5). One possible explanation that could account for the abrogation of RABV growth on Vero and E6 cells at temperatures 35°C and above could be that temperature affects the ability of RABV to bind to host cell receptors at higher temperatures, although this hypothesis is somewhat negated by the fact that RABV was capable of infecting and replicating on mosquito cell culture at 37°C. A second, but more complex, explanation involves the response of mammalian cells to sub-physiological temperatures. Sub-physiological temperatures have been reported to induce the expression of 20 or more genes not induced in cells at 37°C [31,32]. Low temperature cultivation of mammalian cells also results in prolonged generation time and maintenance of cell viability for longer periods, reduced glucose and glutamine consumption, suppressed release of waste products, delayed apoptosis, reduced protease activity, and improved tolerance to shear stress [33-36]. From this, we postulate that changes associated with the response to sub-physiological temperatures in mammalian cells make them susceptible to RABV infection. This is consistent with the fact that RABV could infect mosquito cell culture regardless of temperature, and it stands to reason that poikilotherm cells are more resistant to temperature-induced change because, in nature, they constantly have to deal with shifts in temperature.

## Conclusions

Regardless of the mechanism, understanding the factors involved in the maintenance and spread of an infectious organism are crucial for timely recognition of emerging infections. This becomes especially relevant when one considers that a large proportion of emerging viral infections are caused by multi-host zoonotic RNA viruses, because these viruses have a higher propensity to switch hosts [37-40]. Clearly, RABV is capable of replicating in vertebrate cells if conditions are appropriate, and it has not yet been excluded that RABV might circulate and be amplified in certain vertebrates, e.g., herptiles. Therefore, RABV provides a powerful tool to study the evolution and molecular determinants of flavivirus host range, as well as genetic changes in the pathogen that facilitate emergence. Identifying the mechanism(s) involved in mediating RABV temperature sensitivity will be the subject of future investigations. As such, reverse genetic tools currently are being developed for RABV and will be especially valuable in efforts to elucidate genetic changes that facilitate host switching, which will provide a comprehensive molecular portrait of flavivirus-host cell interactions.

## Competing interests

No competing financial interests exist.

## Authors’ contributions

MTA performed all experiments, conceived and participated in the design of the study, analyzed the data, and drafted the manuscript. LDK conceived the study and participated in its design and coordination and helped draft the manuscript. Both authors read and approved the final manuscript.
